# Shotgun metaproteomics reveals habitat-specific antimicrobial resistance-associated proteins of <em>Escherichia</em> spp. and <em>Salmonella</em> spp. in the gut resistome of free-living long-tailed macaques in Thailand

**DOI:** 10.14202/vetworld.2026.2971-2990

**Published:** 2026-07-14

**Authors:** Wirasak Fungfuang, Daraka Tongthainan, Sawanya Charoenlappanit, Narumon Phaonakrop, Sittiruk Roytrakul, Kongphop Parunyakul

**Affiliations:** 1 Department of Zoology, Faculty of Science, Kasetsart University, Bangkok, Thailand.; 2 Faculty of Veterinary Medicine, Rajamangala University of Technology Tawan-ok, Chonburi, Thailand.; 3 National Center for Genetic Engineering and Biotechnology (BIOTEC), National Science and Technology Development Agency, Pathum Thani, Thailand.; 4 Department of Biology, Faculty of Science, Burapha University, Chonburi, Thailand.

**Keywords:** antimicrobial resistance, Escherichia spp, gut microbiota, long-tailed macaques, metaproteomics, One Health, resistome, Salmonella spp

## Abstract

**Background and Aim:**

Antimicrobial resistance (AMR) is a growing global health concern affecting humans, animals, and the environment within the One Health framework. Free-living non-human primates may act as reservoirs and disseminators of antimicrobial-resistant microorganisms through close interactions with humans and shared environments. This study aimed to characterize habitat-specific AMR-associated proteins of *Escherichia* spp. and *Salmonella* spp. in the gut resistome of free-living long-tailed macaques (*Macaca fascicularis*) inhabiting distinct ecological settings in Thailand using a shotgun metaproteomics approach.

**Materials and Methods:**

A total of 54 fecal samples were non-invasively collected from free-living long-tailed macaques at two locations in Thailand between January and February 2025: Chongkrachok Mountain, Prachuap Khiri Khan Province (natural ecotourism habitat; n = 16) and Phromawat Temple, Chonburi Province (urban-proximate habitat; n = 38). Proteins were extracted and analyzed using liquid chromatography–tandem mass spectrometry. Label-free shotgun metaproteomics was performed using MaxQuant and Perseus platforms. Differential protein expression, functional annotation, and pathway analyses were conducted using MetaboAnalyst 6.0, Gene Ontology classification, and Kyoto Encyclopedia of Genes and Genomes pathway mapping.

**Results:**

Distinct protein expression profiles of *Escherichia* spp. and *Salmonella* spp. were observed between habitats. In *Escherichia* spp., 1,299 proteins were differentially expressed, whereas 231 differentially expressed proteins were identified in *Salmonella* spp. Functional enrichment analysis indicated that proteins associated with microbial proliferation predominated in *Escherichia* spp., while DNA repair and stress-response proteins were enriched in *Salmonella* spp. from the ecotourism habitat. Notably, AMR-associated proteins were substantially more abundant in *Escherichia* spp. from the urban-proximate habitat, where 25 resistance-related proteins were identified compared with six in the ecotourism habitat. Kyoto Encyclopedia of Genes and Genomes pathway analysis revealed enhanced β-lactam resistance mechanisms involving multidrug efflux systems and β-lactamases in the urban-proximate population, whereas TolC-mediated efflux activity predominated in the ecotourism population.

**Conclusion:**

This shotgun metaproteomic analysis provides the first direct evidence of differentially expressed AMR-associated proteins varying by habitat conditions, revealing active functional mechanisms under anthropogenic pressure and geographic location. Our findings suggest that environmental shifts and dietary habits could directly affect AMR-related functions of the gut microbiota of animals. This finding contributes to understanding the dynamics of AMR under the One Health framework and may inform strategies to reduce zoonotic and environmental transmission risks.

## INTRODUCTION

Antimicrobial resistance (AMR) has become a major public health threat in the 21st century [1]. Drug-resistant infections were associated with more than 4.95 million deaths globally in 2019 and are projected to cause up to 10 million deaths annually by 2050 if effective mitigation strategies are not implemented [2]. AMR is a multifaceted challenge driven by interactions among humans, animals, and the environment under the One Health framework. Increasing territorial overlap between humans and wildlife has intensified interactions with free-living animals, including those associated with tourism-related wildlife feeding activities [3, 4]. Previous studies have shown that human-associated factors, including captivity, dietary modification, and other anthropogenic influences, can alter the diversity and composition of the gut microbiome in primates [5]. Ecotourism activities such as feeding, petting, and photography further reduce the natural separation between humans and free-living macaques, potentially increasing the risk of pathogen transmission, including antimicrobial-resistant microorganisms [6]. However, the effects of feeding practices, habitat variation, and wildlife ecology on gut microbiota composition and AMR dynamics remain incompletely understood.

The gut microbiota comprises diverse microorganisms inhabiting the gastrointestinal tract and plays essential roles in host physiology through nutrient metabolism, xenobiotic and drug metabolism, immunomo-dulation, and protection against pathogens [7, 8]. Antimicrobial use, together with host- and environment-related factors such as habitat characteristics and dietary composition, can exert selective pressure on gut microbial communities, promoting the persistence and expansion of antimicrobial-resistant bacteria [9]. Furthermore, metagenomic and binning analyses have demonstrated that anthropogenic pressures increase the abundance of dominant AMR gene hosts and facilitate the emergence and dissemination of multidrug- and β-lactam-resistant microorganisms across environmental gradients [10]. *E. coli* and *Salmonella* spp. are among the most important zoonotic bacterial pathogens associated with foodborne illnesses worldwide, and resistance in both groups has increased substantially in recent years [11, 12]. *Escherichia* spp., common gut commensals in humans and macaques, are widely used as model organisms for investigating bacterial pathogenesis and epidemiological transmission. Importantly, commensal *E. coli* may serve as reservoirs of AMR and virulence genes that can be transmitted between humans and animals through the fecal–oral route [13, 14]. In contrast, *Salmonella* spp. are major foodborne pathogens commonly transmitted through fecally contaminated food and water [15–17].

Long-tailed macaques (*Macaca fascicularis*) are non-human primates (NHPs) widely distributed throughout Thailand [18]. These animals are frequently exposed to human activities through tourism, urbanization, and increasing habitat encroachment, resulting in close interactions among macaques, humans, livestock, and domestic animals within shared environments. Such interactions often contribute to human–macaque conflict and may facilitate the exchange of microorganisms and AMR determinants. Previous metagenomic studies have characterized resistome diversity in wild and captive macaques [19], demonstrating greater ARG diversity in semi-captive populations and substantial alterations in the gut microbiota of Thai long-tailed macaques exposed to captivity or anthropogenic feeding [20]. Nevertheless, these studies primarily identify the presence of resistance genes and do not determine whether such genes are actively expressed at the protein level.

Despite increasing interest in wildlife-associated AMR, significant knowledge gaps remain regarding the functional expression of resistance mechanisms within the gut microbiota of free-living NHPs. Most available studies have relied on culture-based methods, metagenomics, or resistome profiling approaches that identify resistance genes but cannot determine their translation into biologically active proteins. Consequently, the actual resistance mechanisms operating within wildlife gut ecosystems remain poorly understood. Moreover, information on how habitat characteristics, anthropogenic pressures, dietary differences, and geographic variation influence the expression of AMR-associated proteins in *Escherichia* spp. and *Salmonella* spp. is particularly limited. To date, no study has applied shotgun metaproteomics to characterize active AMR-related protein expression in free-living *M. fascicularis* inhabiting ecologically distinct environments in Thailand. Addressing this gap is essential for understanding the functional ecology of wildlife resistomes and clarifying the role of free-living macaques as potential reservoirs and disseminators of antimicrobial-resistant microorganisms at the human–wildlife interface.

Therefore, this study aimed to characterize habitat-specific AMR-associated protein expression in *Escherichia* spp. and *Salmonella* spp. within the gut resistome of free-living long-tailed macaques inhabiting two ecologically distinct environments in Thailand using a shotgun metaproteomics approach. Specifically, we sought to identify differentially expressed proteins, determine their functional annotations, and evaluate AMR-associated pathways in macaque populations exposed to varying degrees of anthropogenic influence. Metaproteomics provides direct insights into microbial functional activity and enables the identification of actively expressed resistance mechanisms beyond the mere presence of genes [21]. We hypothesized that macaques inhabiting urban-proximate environments would exhibit greater expression and diversity of AMR-associated proteins than macaques in natural ecotourism habitats due to increased exposure to anthropogenic pressures. The findings of this study provide novel insights into the functional ecology of wildlife-associated AMR, improve understanding of habitat-driven variation in gut resistomes, and support the development of evidence-based surveillance and mitigation strategies within a One Health framework.

## MATERIALS AND METHODS

### Ethical approval

This study was reviewed and approved by the Animal Care and Use Ethics Committee (ACUC), Faculty of Veterinary Medicine, Rajamangala University of Technology Tawan-ok, Thailand, under approval reference number RMUTTO-ACUC-2-2023-010.

The study was conducted in accordance with the institutional guidelines for the ethical use of animals in research and complied with internationally accepted principles for wildlife research and animal welfare. Free-living long-tailed macaques (*Macaca fascicularis*) were not captured, restrained, sedated, anesthetized, marked, or experimentally manipulated at any stage of the investigation. Fecal samples were collected exclusively through non-invasive sampling immediately after natural defecation to avoid disturbance, stress, or injury to the animals. Collection was performed at the center of freshly voided feces using sterile sampling equipment, while minimizing environmental contamination and avoiding repeated sampling from the same individual, as observed in the field.

All field activities were conducted with minimal interference to the animals' normal behavior and habitat. No food was provided, and no procedures were undertaken that could alter animal movement, social interactions, or foraging behavior. Sample transportation, storage, and laboratory processing were conducted in accordance with established biosafety and biosecurity procedures to ensure personnel safety and the integrity of biological specimens.

This non-invasive study involved only observational fieldwork and fecal sample collection from naturally occurring wildlife populations; therefore, no invasive procedures, euthanasia, or experimental interventions were performed. All efforts were made to ensure animal welfare and minimize potential impacts on free-living macaque populations and their natural environment.

### Study period and location

The study was conducted from January to February 2025 at two geographically distinct locations in Thailand, selected for differences in habitat characteristics, food availability, and the degree of human–macaque interaction. The first study site was Chongkrachok Mountain, Mueang Prachuap Khiri Khan District, Prachuap Khiri Khan Province (11°48'55.77"N, 99°47'54.00"E), representing a natural habitat with ecotourism activities and supplementary feeding by visitors (location P). The second study site was Phromawat Temple in Si Racha District, Chonburi Province (13°10'32.03"N, 100°57'08.16"E), representing an urban-proximate habitat characterized by frequent human–macaque interactions, tourism, and religious gatherings (location S).

**Figure 1:**
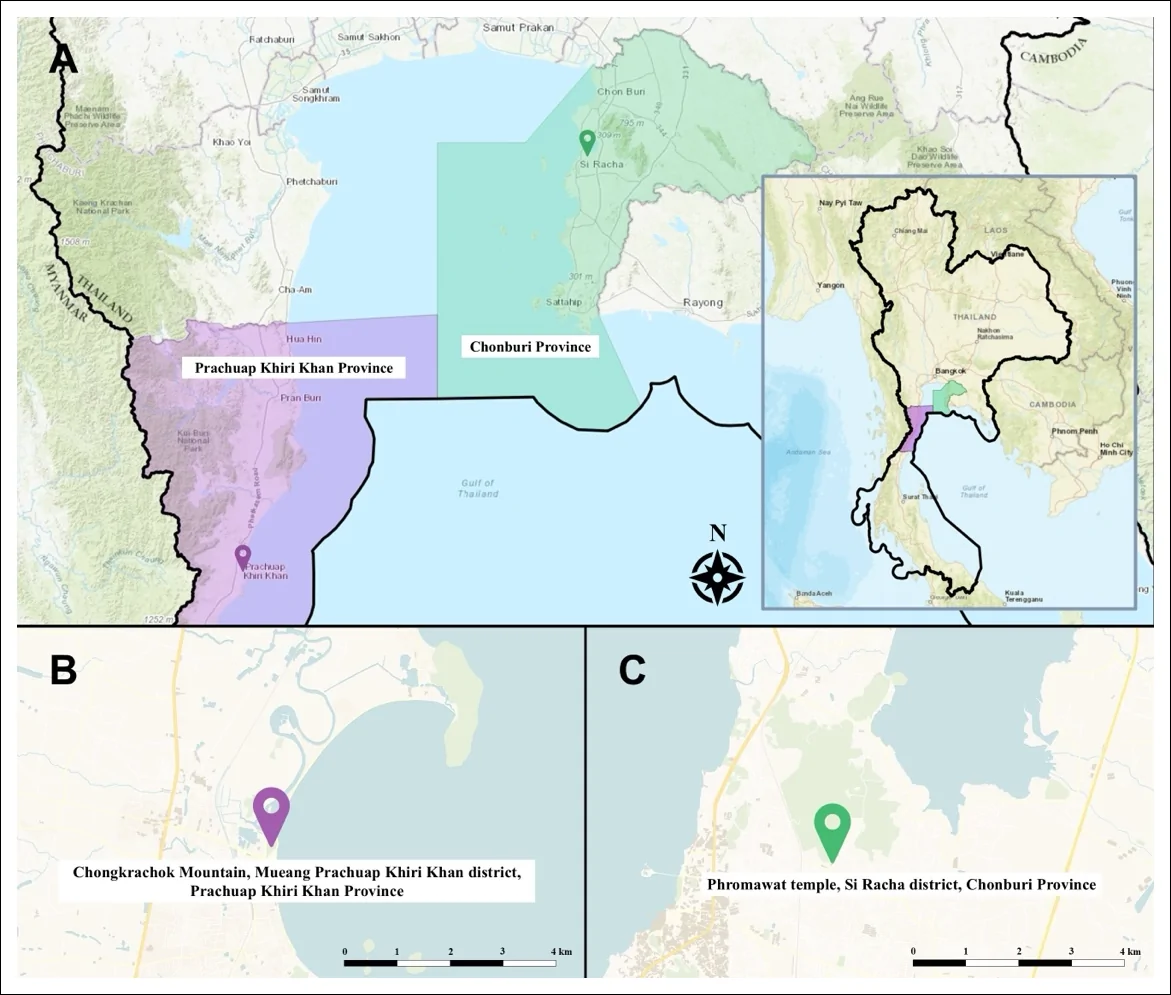
Map of the sampling locations of free-living long-tailed macaques (*Macaca fascicularis*) from two distinct habitats in Thailand: Chongkrachok Mountain, Mueang Prachuap Khiri Khan District, Prachuap Khiri Khan Province (11°48'55.77"N, 99°47'54.00"E) and Phromawat Temple, Si Racha District, Chonburi Province (13°10'32.03"N, 100°57'08.16"E). The map was generated using OSM data and QGIS version 4.0.1.

As shown in Figure 1, the study sites were selected to represent contrasting ecological conditions and varying levels of anthropogenic influence on free-living *M. fascicularis* populations. These habitats are typical environments inhabited by long-tailed macaques in Thailand, including temples, community forests, public parks, and nature-based ecotourism areas.

### Study design

A cross-sectional comparative metaproteomic study was conducted to investigate habitat-associated differences in the gut resistome of free-living long-tailed macaques. A total of 54 fecal samples were collected and categorized according to sampling location. Group P consisted of samples collected from Chongkrachok Mountain (n = 16), whereas Group S consisted of samples collected from Phromawat Temple (n = 38). Comparative analyses were subsequently performed to evaluate differences in protein expression profiles, AMR-associated proteins, and functional pathways between the two populations.

### Fecal sample collection

Fresh fecal samples were non-invasively collected from free-living long-tailed macaque colonies inhabiting natural environments adjacent to human communities and tourist attractions. Sample collection was performed immediately after defecation to minimize the possibility of repeated sampling from the same individual. Fecal material was collected from the center of each stool mass using sterile scoops and transferred into sterile fecal collection tubes to minimize contamination from soil, substrate, and other environmental sources.

The sex and age of individual macaques could not be accurately determined because samples were collected after direct observation of defecation without animal capture or identification. Based on field observations, the sampled animals likely included adult males and females. During transportation, samples were stored in insulated containers containing ice packs and subsequently preserved at −80°C until laboratory analysis.

### Protein extraction and quantification

Frozen fecal samples were processed by adding phosphate-buffered saline (pH 7.2), then vortexed and centrifuged at 2,000 × *g* for 20 min at 4°C. The resulting supernatants were collected and stored at −80°C until further analysis.

Fecal supernatants were mixed with acetone at a ratio of 2:1 (v/v) and centrifuged at 10,000 × *g *for 10 min. The resulting pellets were suspended in lysis buffer containing 0.25% (w/v) sodium dodecyl sulfate and 50 mM Tris-HCl (pH 9.0). Protein concentrations were determined using the Lowry method [22] with bovine serum albumin as the reference standard. Pooled samples for each location group were prepared by combining equal amounts of protein from individual samples. All analyses were performed in triplicate.

For protein digestion, 5 μg of protein from each sample was reduced using dithiothreitol and subsequently alkylated with iodoacetamide. Samples were then digested with sequencing-grade trypsin (Promega, Mannheim, Germany) at a ratio of 1:20 and incubated overnight at 37°C.

### Liquid chromatography–tandem mass spectrometry (LC-MS/MS)

Protein expression profiles were determined using LC-MS/MS. Digested peptide samples were dried, reconstituted in 0.1% formic acid, and injected into an Ultimate 3000 Nano/Capillary LC system (Thermo Scientific, Loughborough, UK) coupled to a Hybrid Quadrupole Time-of-Flight Impact II mass spectrometer (Bruker Daltonics, Bremen, Germany) equipped with a CaptiveSpray ion source.

One microliter of each peptide digest was loaded onto a μ-Precolumn (300 μm internal diameter × 5 mm, C18 PepMap 100, 5 μm, 100 Å; Thermo Fisher Scientific) for online desalting and preconcentration. Peptide separation was performed using an analytical column (75 μm internal diameter × 15 cm, Acclaim PepMap C18, 2 μm, 100 Å; Thermo Fisher Scientific) maintained at 60°C.

The mobile phases consisted of 0.1% formic acid in water (Solvent A) and 0.1% formic acid in 80% acetonitrile (Solvent B). Peptides were eluted using a linear gradient of 5%–55% Solvent B over 30 min at a flow rate of 0.30 μL/min.

Electrospray ionization was performed using the CaptiveSpray source at 1.6 kV. Nitrogen was used as the drying gas at approximately 50 L/h and as the collision gas. Mass spectra and tandem mass spectra were acquired in positive-ion mode at a frequency of 2 Hz over an m/z range of 150–2,200. Collision energy was optimized to 10 eV for each m/z value. All samples were analyzed in triplicate to ensure reproducibility.

### Protein identification and bioinformatics analysis

A label-free shotgun metaproteomic workflow integrating differential expression analysis and functional annotation was employed to characterize active AMR-associated proteins in *Escherichia* spp. and *Salmonella* spp. Protein identification and label-free quantification were performed using MaxQuant software version 2.5.0.0 (Max Planck Institute of Biochemistry, Martinsried, Germany) with the Andromeda search engine [23].

Mass spectra were searched against the UniProt databases for *Escherichia* and *Salmonella*. MaxQuant parameters were applied, including trypsin specificity with a maximum of two missed cleavages, carbamido-methylation of cysteine as a fixed modification, and oxidation of methionine and N-terminal acetylation as variable modifications. Proteins were identified based on peptides containing at least seven amino acids, with a minimum requirement of two peptides per protein, including at least one unique peptide. Peptide and protein false discovery rates (FDR) were controlled at 1% using a reversed decoy database strategy.

The ProteinGroups.txt output generated by MaxQuant was imported into Perseus software version 2.0.11.0 (Max Planck Institute of Biochemistry, Martinsried, Germany) for downstream analyses [23]. Contaminant proteins were removed before statistical analyses. Protein intensities were log2-transformed, and missing values were imputed using a constant value of zero. Pairwise comparisons between experimental groups were performed using Student's t-test.

Functional annotation of proteins was performed using Gene ontology (GO) terms retrieved from the UniProt database. Statistical analyses, including heatmaps, volcano plots, principal component analysis (PCA), and partial least squares-discriminant analysis (PLS-DA), were performed using MetaboAnalyst 6.0 [24]. Differentially expressed proteins were identified using an FDR-adjusted p < 0.05 and fold change >2 as significance thresholds.

Venn diagrams were generated using Jvenn software (https://jvenn.toulouse.inrae.fr/app/example.html) to compare differentially expressed proteins among study groups [25]. Functional pathway enrichment analysis was performed using the Kyoto Encyclopedia of Genes and Genomes (KEGG) Mapper reconstruction tool based on gene names and the abundance of upregulated and downregulated proteins.

## RESULTS

### Protein expression patterns of *Escherichia* spp. and *Salmonella* spp. in locations P and S

**Figure 2:**
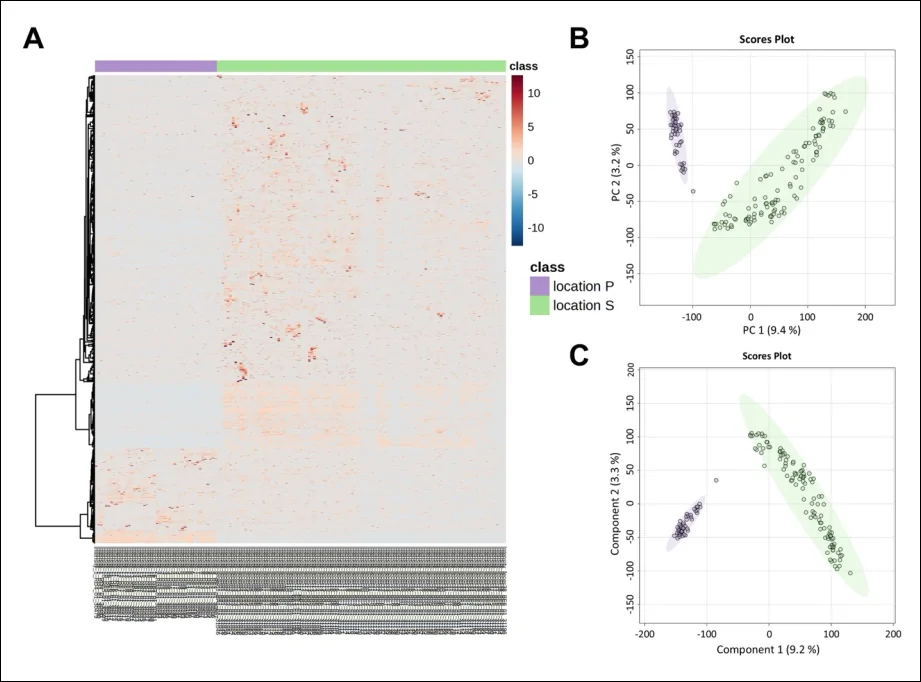
Differential protein expression analysis of *Escherichia* spp. in the fecal microbiomes of long-tailed macaques from locations P and S. (A) Heatmap showing differentially expressed proteins (DEPs) based on Euclidean distance and Ward clustering. Protein abundance is displayed on a normalized scale ranging from blue (low abundance) to red (high abundance). (B) PCA score plot demonstrating separation between the two location groups at the 95% confidence interval. (C) PLS-DA score plot showing covariance between component 1 and component 2 and identifying DEPs between the two locations.

**Figure 3:**
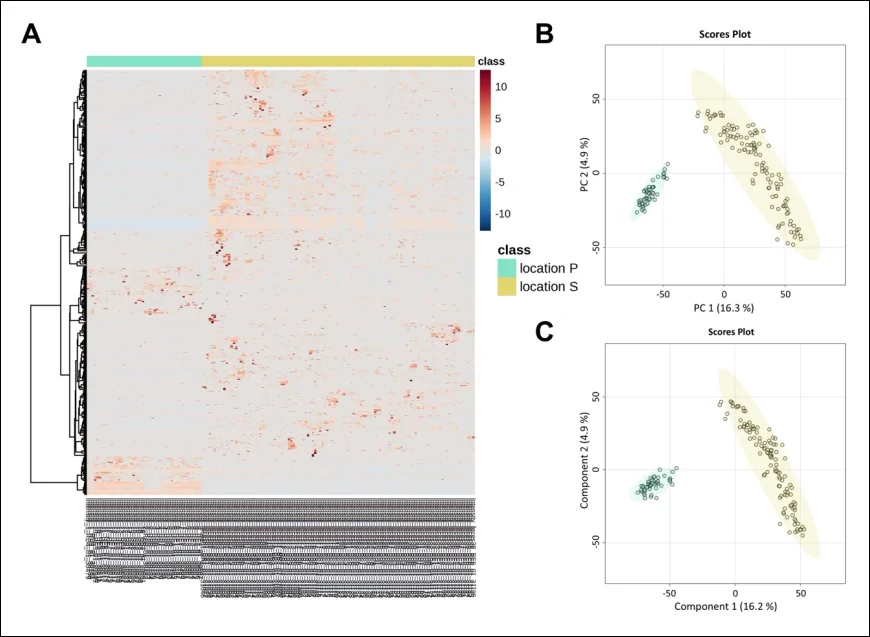
Differential protein expression analysis of *Salmonella* spp. in the fecal microbiomes of long-tailed macaques from locations P and S. (A) Heatmap showing differentially expressed proteins (DEPs) based on Euclidean distance and Ward clustering. Protein abundance is displayed on a normalized scale ranging from blue (low abundance) to red (high abundance). (B) PCA score plot demonstrating separation between the two location groups at the 95% confidence interval. (C) PLS-DA score plot showing covariance between component 1 and component 2 and identifying DEPs between the two locations.

Hierarchical clustering analysis was performed using MetaboAnalyst based on Differentially expressed proteins (DEPs) identified in *Escherichia* spp. (8,989 proteins) and *Salmonella* spp. (1,029 proteins) from the fecal microbiomes of free-living long-tailed macaques (Figures 2A and 3A, respectively). The dendrograms were constructed according to the degree of similarity among expressed proteins to provide an overall visualization of the proteomic profiles. Samples collected from the same location exhibited similar protein expression patterns, whereas clear differences were observed between locations P and S. These findings indicate that the DEP profiles of both *Escherichia* spp. and *Salmonella* spp. can effectively discriminate between the two habitats.

To further compare protein expression patterns between locations P and S, PCA was performed. The PCA score plots demonstrated a clear separation between the two locations along the first principal component (Figure 2B and Figure 3B). To maximize group discrimination, PLS-DA was subsequently performed (Figures 2C and 3C). The PLS-DA results were consistent with the PCA findings and further confirmed distinct proteomic signatures associated with each habitat.

**Functional annotation of DEPs in** *Escherichia* **spp.**

**Figure 4:**
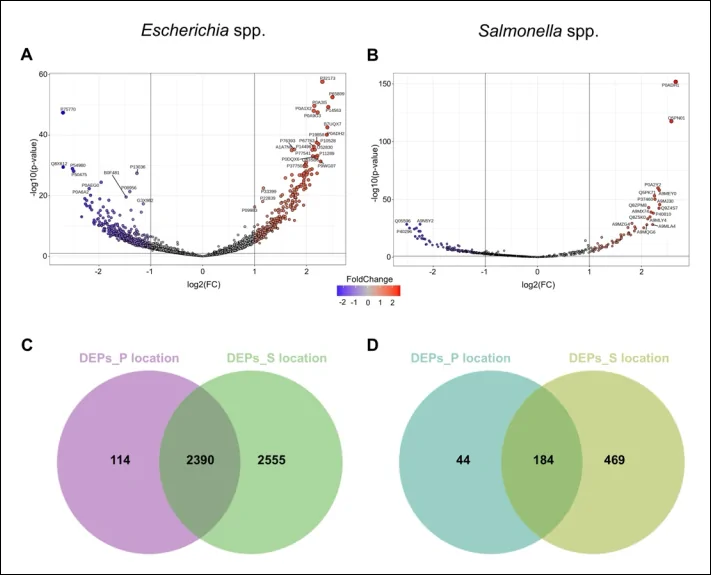
Differential expression profiles of *Escherichia* spp. and *Salmonella* spp. in the fecal microbiomes of long-tailed macaques from locations P and S. (A) Volcano plot showing differentially expressed proteins (DEPs) of *Escherichia* spp. using significance thresholds of p < 0.05 and log₂ fold change (FC) ≥1 or ≤−1. (B) Volcano plot showing DEPs of *Salmonella* spp. using significance thresholds of p < 0.05 and log₂FC ≥1 or ≤−1. (C) Venn diagram illustrating unique and shared DEPs of *Escherichia* spp. between locations P and S. (D) Venn diagram illustrating unique and shared DEPs of *Salmonella* spp. between locations P and S.

Volcano plot analysis using significance thresholds of fold change >2 and p < 0.05 demonstrated substantial differences in protein expression between locations P and S (Figure 4A). A total of 8,989 proteins were detected across both locations, of which 1,299 proteins were differentially expressed. Among these DEPs, 268 proteins were upregulated and 1,031 proteins were downregulated in location P compared with location S. The Venn diagram revealed 2,390 proteins that were differentially expressed across the comparison groups (Figure 4C).

**GO analysis of upregulated proteins in** *Escherichia* **spp.**

**Figure 5:**
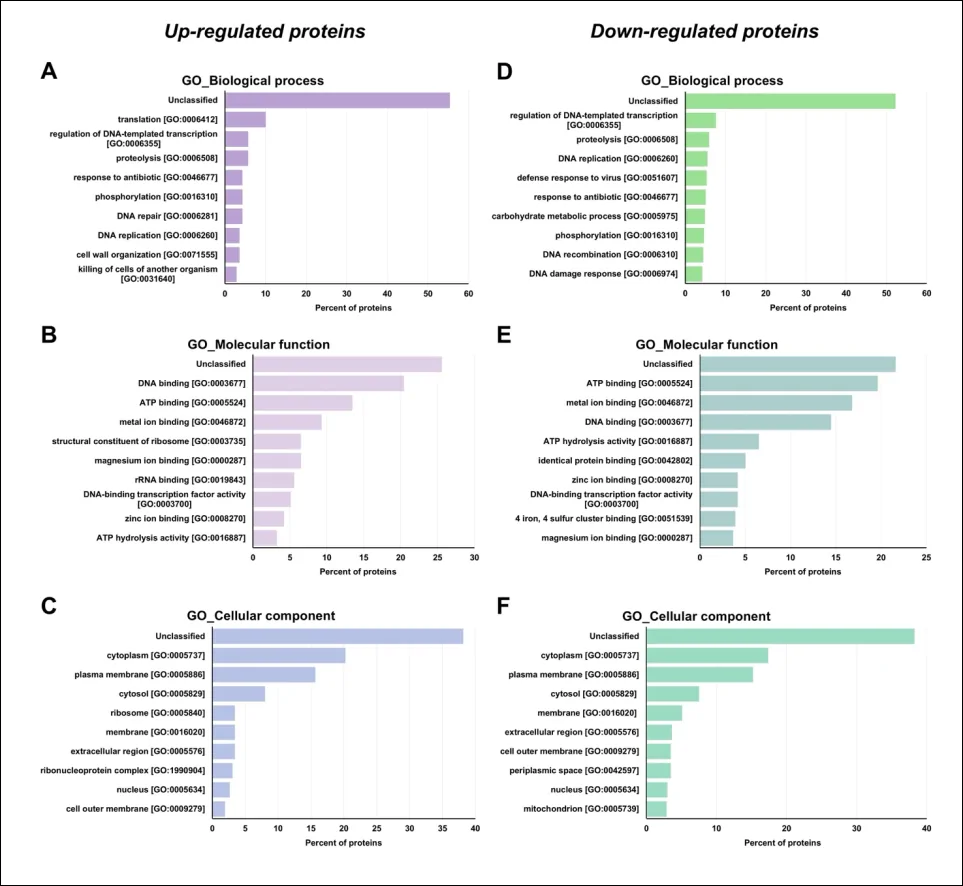
Gene ontology (GO)-based functional annotation of differentially expressed proteins (DEPs) of *Escherichia* spp. in the fecal microbiomes of long-tailed macaques from location P compared with location S. (A) Biological process classification of the 268 upregulated proteins in location P. (B) Molecular function classification of the 268 upregulated proteins in location P. (C) Cellular component classification of the 268 upregulated proteins in location P. (D) Biological process classification of the 1,031 downregulated proteins in the location P versus location S comparison. (E) Molecular function classification of the 1,031 downregulated proteins in the location P versus location S comparison. (F) Cellular component classification of the 1,031 downregulated proteins in the location P versus location S comparison.

GO classification demonstrated that the upregulated proteins in location P were primarily associated with unclassified proteins (77 proteins), translation (14 proteins), regulation of DNA-templated transcription (8 proteins), proteolysis (8 proteins), and response to antibiotics (6 proteins) (Figure 5A).

Among molecular function categories, excluding unclassified proteins, the most abundant functions included DNA binding (44 proteins), adenosine triphosphate (ATP) binding (29 proteins), metal ion binding (20 proteins), structural constituent of ribosome (14 proteins), and magnesium ion binding (14 proteins) (Figure 5B).

Among cellular components, the majority of proteins were localized to the cytoplasm (53 proteins), followed by the plasma membrane (41 proteins), the cytosol (21 proteins), ribosomes (9 proteins), and the extracellular region (9 proteins) (Figure 5C).

**GO analysis of downregulated proteins in** *Escherichia* **spp.**

Among the downregulated proteins identified in location P relative to location S, the predominant biological process categories included unclassified proteins (246 proteins), regulation of DNA-templated transcription (36 proteins), proteolysis (28 proteins), DNA replication (26 proteins), defense response to virus (25 proteins), and response to antibiotics (24 proteins) (Figure 5D).

Within the molecular function category, ATP-binding (n = 160), metal ion binding (n = 137), DNA binding (n = 118), and ATP hydrolysis activity (n = 53) were the most represented functions (Figure 5E).

Based on cellular component classification, the majority of proteins were categorized as unclassified proteins (n = 397), cytoplasm (n = 180), and plasma membrane (n = 158) (Figure 5F).

**Functional annotation of DEPs in** *Salmonella* **spp.**

Volcano plot analysis identified 1,029 proteins in *Salmonella* spp. from locations P and S. Among these, 66 DEPs were upregulated and 165 were downregulated in location P compared with location S. The Venn diagram showed 184 proteins commonly differentially expressed between the two groups (Figure 4D).

**GO analysis of upregulated proteins in** *Salmonella* **spp.**

**Figure 6:**
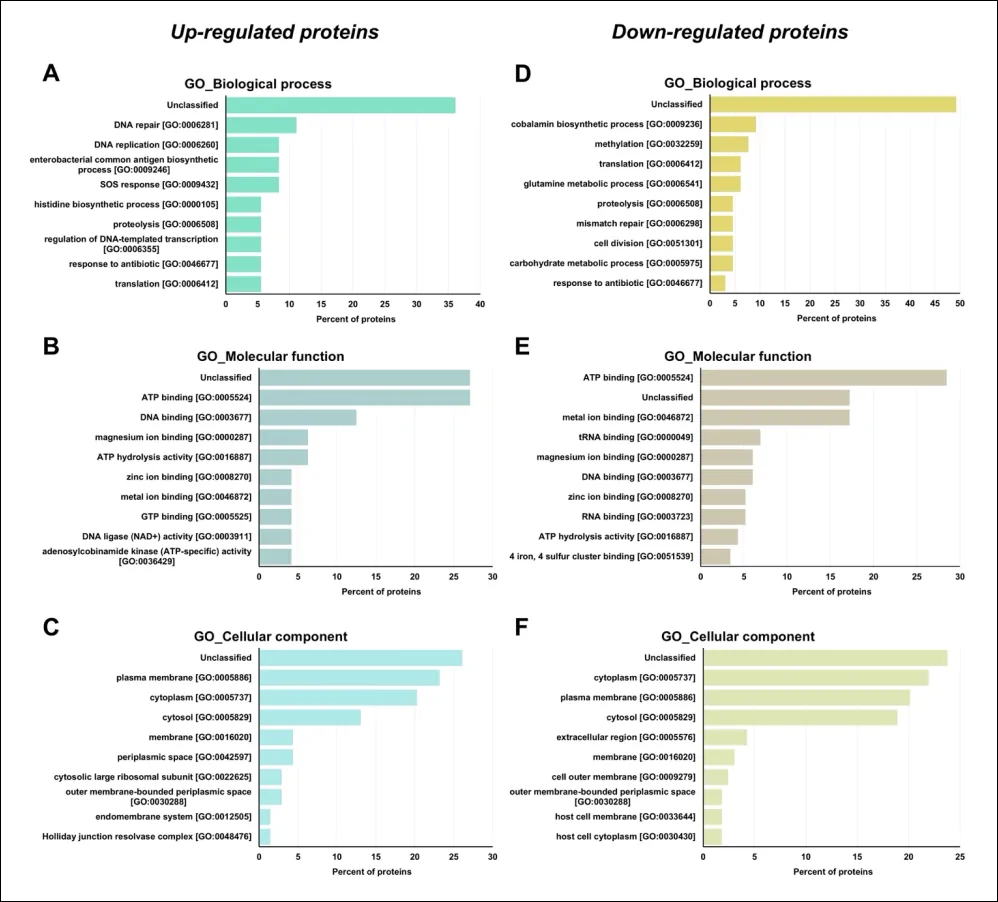
Gene ontology (GO)-based functional annotation of differentially expressed proteins (DEPs) of *Salmonella* spp. in the fecal microbiomes of long-tailed macaques from location P compared with location S. (A) Biological process classification of the 66 upregulated proteins in location P. (B) Molecular function classification of the 66 upregulated proteins in location P. (C) Cellular component classification of the 66 upregulated proteins in location P. (D) Biological process classification of the 165 downregulated proteins in the location P versus location S comparison. (E) Molecular function classification of the 165 downregulated proteins in the location P versus location S comparison. (F) Cellular component classification of the 165 downregulated proteins in the location P versus location S comparison.

The upregulated proteins in location P were mainly associated with unclassified proteins (n = 13), DNA repair (n = 4), SOS response (n = 3), enterobacterial common antigen biosynthetic process (n = 3), and DNA replication (n = 3) (Figure 6A).

The predominant molecular functions included ATP-binding (n = 13), DNA binding (n = 6), ATP hydrolysis activity (n = 3), and magnesium ion binding (n = 3) (Figure 6B).

The principal cellular components associated with upregulated proteins were the plasma membrane (n = 16) and cytoplasm (n = 14) (Figure 6C).

**GO analysis of downregulated proteins in** *Salmonella* **spp.**

The downregulated proteins were primarily involved in unclassified proteins (32 proteins), cobalamin biosynthetic process (n = 6), methylation (n = 5), translation (n = 4), and glutamine metabolic process (n = 4) (Figure 6D).

ATP-binding (n = 33) represented the most abundant molecular function category, followed by unclassified proteins (n = 20), metal ion binding (n = 20), and tRNA binding (n = 8) (Figure 6E).

The predominant cellular components included unclassified proteins (n = 39), cytoplasm (n = 36), plasma membrane (n = 33), cytosol (n = 31), and extracellular region (n = 7) (Figure 6F).

**Antibiotic resistance-associated proteins in** *Escherichia* **spp. and** *Salmonella* **spp.**

Among the 268 upregulated DEPs of *Escherichia* spp. in location P, six proteins were associated with antibiotic response activity, including multidrug resistance (MDR) protein (P39386), chloramphenicol acetyltransferase 3 (P00484), outer membrane protein TolC (P02930), undecaprenyl-phosphate 4-deoxy-4-formamido-L-arabinose transferase (B7LM77), primosomal protein N′ (P17888), and pentapeptide repeat protein QnrB4 (Q2PT27).

Among the 1,031 downregulated DEPs in location P (corresponding to proteins upregulated in location S), 25 proteins were associated with antibiotic resistance, including aminoglycoside adenylyltransferase (P0AG05), penicillin-binding protein 1C (P76577), probable aminoglycoside efflux pump (P24177), putative O-antigen transporter (P37746), and penicillin G acylase (P06875). Detailed information is presented in Tables 1 and 2.

**Table 1:** Upregulated antimicrobial resistance-associated proteins of Escherichia spp. identified in the fecal microbiomes of long-tailed macaques from location P compared with location S.

**Protein ID**	**Protein name**	**Peptide sequence**	**Fold change**	**p-value**	**Gene ontology (GO) – Biological process**
P39386	Multidrug resistance protein MdtM	FTLFSNK	3.4288	1.85 × 10⁻¹⁶	Response to antibiotic; bile acid and bile salt transport; potassium ion export across plasma membrane; sodium ion export across plasma membrane; regulation of cellular pH; xenobiotic detoxification by transmembrane export across the plasma membrane
P00484	Chloramphenicol acetyltransferase 3 (EC 2.3.1.28)	EHFEFYRHR	3.0014	9.59 × 10⁻¹⁴	Response to antibiotic
P02930	Outer membrane protein TolC (multidrug efflux pump subunit TolC)	GAAGTQYDDSNMGQNK	2.6668	3.38 × 10⁻¹⁰	Response to antibiotic; bile acid and bile salt transport; enterobactin transport; monoatomic ion transmembrane transport; response to organic cyclic compound; response to toxic substance; response to xenobiotic stimulus; xenobiotic detoxification by transmembrane export across the cell outer membrane; xenobiotic detoxification by transmembrane export across the plasma membrane
B7LM77	Undecaprenyl-phosphate 4-deoxy-4-formamido-L-arabinose transferase (EC 2.4.2.53)	ADEGYDVVGTVR	2.6410	1.31 × 10⁻¹⁰	4-Amino-4-deoxy-α-L-arabinopyranosyl undecaprenyl-phosphate biosynthetic process; lipid A biosynthetic process; lipopolysaccharide biosynthetic process; response to antibiotic
P17888	Primosomal protein N′ (ATP-dependent helicase PriA; replication factor Y)	AHSEQIPIILGSATPALETLCNVQQKKYR	2.4961	4.76 × 10⁻¹³	DNA recombination; DNA replication; DNA replication initiation; DNA replication, synthesis of RNA primer; DNA unwinding involved in DNA replication; DNA-templated DNA replication; double-strand break repair; plasmid maintenance; replication fork processing; response to antibiotic; response to gamma radiation
Q2PT27	Pentapeptide repeat protein QnrB4 (plasmid-mediated quinolone resistance determinant)	DAIFKSCDLSMADFRNINALGIEIR	2.4381	9.81 × 10⁻¹⁰	Response to antibiotic

Location P = Chongkrachok Mountain, Prachuap Khiri Khan Province; Location S = Phromawat Temple, Chonburi Province. GO = Gene Ontology. Fold change >2 and p < 0.05 were considered significant. Protein names and annotations were obtained from UniProt and GO databases.

For *Salmonella* spp., two of the 66 upregulated DEPs in location P were associated with antibiotic response activity, namely signal transduction protein PmrD (P37589) and aminoglycoside N(6′)-acetyltransferase type 1 (Q9R381). In contrast, two downregulated proteins associated with antibiotic resistance were aminoglycoside N(3)-acetyltransferase III (P0A255) and MDR protein MdtM (Q8XFG0). Detailed results are presented in Table 3.

**KEGG pathway analysis of AMR-associated proteins in** *Escherichia* **spp.**

**Figure 7:**
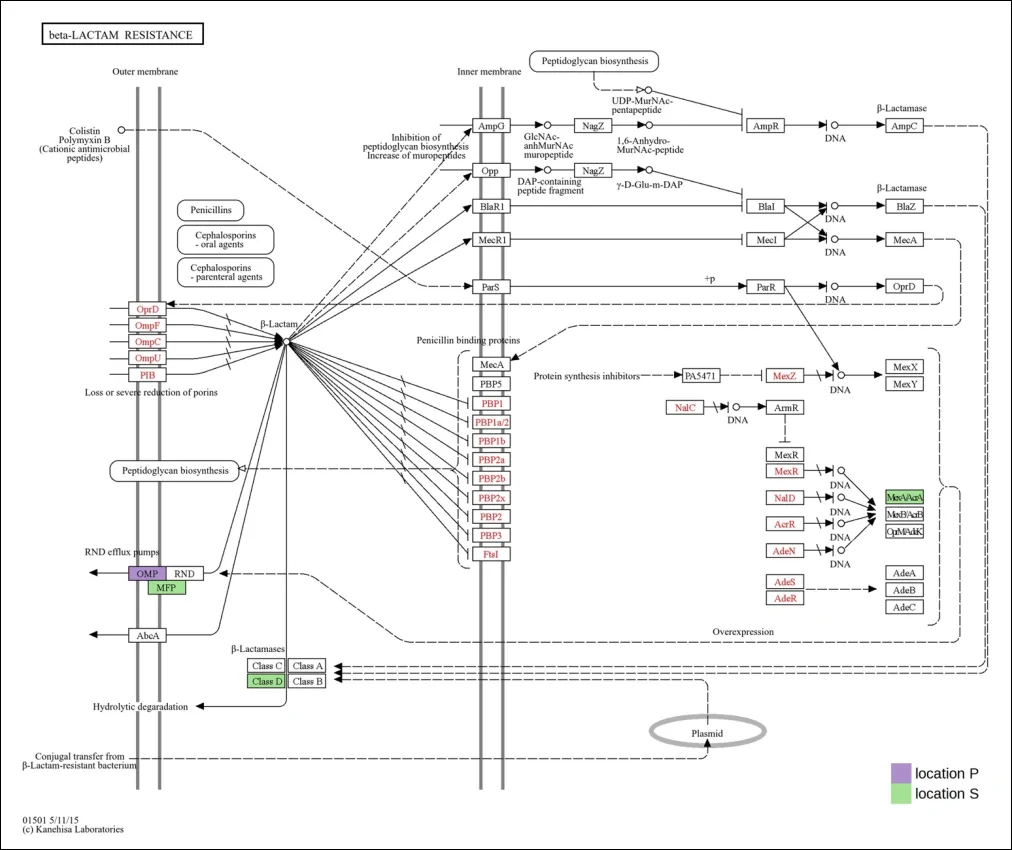
KEGG pathway analysis of antimicrobial resistance-associated proteins of *Escherichia* spp. in the fecal microbiomes of long-tailed macaques. Purple and green indicate proteins upregulated in locations P and S, respectively. The analysis highlights proteins enriched in the β-lactam resistance pathway and associated antimicrobial resistance mechanisms.

The AMR-associated proteins identified in *Escherichia* spp. from locations P and S were further analyzed using KEGG pathway mapping to investigate potential resistance mechanisms (Figure 7).

**Table 2:** Downregulated antimicrobial resistance-associated proteins of Escherichia spp. identified in the fecal microbiomes of long-tailed macaques from location P compared with location S.

**Protein ID**	**Protein name**	**Peptide sequence**	**Fold change**	**p-value**	**Gene ontology (GO) – Biological process**
P0AG05	Aminoglycoside (3'') (9) adenylyltransferase (EC 2.7.7.47)	ALINDLLETSASPGESEILR	0.49454	0.000633	Response to antibiotic
P76577	Penicillin-binding protein 1C (PBP-1c)	ESREEPIWLAPR	0.49436	0.000174	Cell wall organization; peptidoglycan biosynthetic process; positive regulation of cell division; proteolysis; regulation of cell shape; response to antibiotic
P24177	Probable aminoglycoside efflux pump (acriflavine resistance protein D)	DGGMVPFSAFATSR	0.49019	0.000805	Bile acid and bile salt transport; response to antibiotic; response to toxic substance; xenobiotic detoxification by transmembrane export across the cell outer membrane; xenobiotic transport
P37746	Putative O-antigen transporter	GVILIKK	0.48711	0.000376	DNA damage response; O-antigen biosynthetic process; response to antibiotic
P06875	Penicillin G acylase (EC 3.5.1.11)	EVASLLAWTHQMK	0.48028	0.000264	Antibiotic biosynthetic process; response to antibiotic
P0A1V9	Beta-lactamase OXA-2 (EC 3.5.2.6)	AMLVFDPVR	0.45951	0.000139	Antibiotic catabolic process; response to antibiotic
P9WIE5	Catalase-peroxidase (EC 1.11.1.21)	AAGHNITVPFTPGR	0.45270	1.99 × 10⁻⁵	Cellular response to hydrogen peroxide; hydrogen peroxide catabolic process; positive regulation of DNA repair; response to antibiotic; response to oxidative stress
P20831	DNA gyrase subunit A (EC 5.6.2.2)	AYETGRGSIQMRSR	0.43548	2.91 × 10⁻⁵	DNA negative supercoiling; DNA topological change; DNA-templated DNA replication; response to antibiotic
Q83QT8	Bifunctional polymyxin resistance protein ArnA	AFYGSVAHLAAER	0.42836	3.86 × 10⁻⁵	Lipid A biosynthetic process; lipopolysaccharide biosynthetic process; response to antibiotic
P00809	Beta-lactamase 1 (EC 3.5.2.6)	EDLVDYSPVTEK	0.42080	3.77 × 10⁻⁵	Beta-lactam antibiotic catabolic process; response to antibiotic
P62577	Chloramphenicol acetyltransferase (EC 2.3.1.28)	FYPAFIHILAR	0.42028	3.24 × 10⁻⁵	Response to antibiotic
P04382	Dihydrofolate reductase (EC 1.5.1.3)	EMVETHWYKIDEVTTLTESVYK	0.42018	3.17 × 10⁻⁵	One-carbon metabolic process; response to antibiotic; response to methotrexate; tetrahydrofolate biosynthetic process
P0C2H3	Macrolide export ATP-binding/permease protein MacB 2 (EC 7.6.2.-)	DDRNALQEVIIDENTR	0.39930	2.33 × 10⁻⁵	Response to antibiotic
Q05053	Acylase ACY 1 proenzyme (includes cephalosporin acylase)	ATADMYECLSDEIGK	0.39840	1.65 × 10⁻⁵	Glutathione catabolic process; response to antibiotic
P27245	Multiple antibiotic resistance protein MarR	LTTGGAAICEQCHQLVGQDLHQELTK	0.39554	1.27 × 10⁻⁵	Cellular response to antibiotic; negative regulation of DNA-templated transcription; regulation of DNA-templated transcription; response to heat
P35695	Beta-lactamase OXA-7 (EC 3.5.2.6)	EVGEVRMQKYLK	0.37483	4.58 × 10⁻⁶	Antibiotic catabolic process; response to antibiotic
P13364	DNA gyrase subunit B (EC 5.6.2.2)	AFVEYLNTNKTPVNSQVFHFSVQR	0.36280	7.80 × 10⁻⁶	DNA topological change; DNA-templated DNA replication; response to antibiotic
P0AES5	DNA gyrase subunit A (EC 5.6.2.2)	AEVEVDAKTGR	0.35809	9.17 × 10⁻⁸	DNA negative supercoiling; DNA topological change; DNA-templated DNA replication; response to antibiotic
P20083	DNA topoisomerase IV subunit B (EC 5.6.2.2)	CAYVLSVKMQDPQFAGQTK	0.35549	3.50 × 10⁻⁸	Chromosome organization; chromosome segregation; DNA topological change; plasmid partitioning; response to antibiotic; sister chromatid cohesion
Q83P87	Multidrug resistance outer membrane protein MdtP	AFFGLDAIHLDTLFK	0.34791	1.59 × 10⁻⁶	Response to antibiotic
P27303	Multidrug export protein EmrA	IISPMTGYVSR	0.33738	5.41 × 10⁻⁸	Response to antibiotic; response to toxic substance; xenobiotic detoxification by transmembrane export across the cell outer membrane; xenobiotic detoxification by transmembrane export across the plasma membrane
P33941	ABC transporter ATP-binding/permease protein YojI	AEYVFNNLYIPDAQEYRHHIIR	0.32348	1.31 × 10⁻⁷	Microcin transport; response to antibiotic
P0AE07	Multidrug efflux pump subunit AcrA	AIFPNPDHTLLPGMFVR	0.31887	1.43 × 10⁻⁶	Response to antibiotic; response to toxic substance
Q59397	Dihydrofolate reductase type 9 (EC 1.5.1.3)	KTFASLPKVLPGR	0.31394	2.80 × 10⁻⁸	Glycine biosynthetic process; one-carbon metabolic process; response to antibiotic
P23874	Serine/threonine-protein kinase toxin HipA (EC 2.7.11.1)	LANGAHTFK	0.31063	3.29 × 10⁻⁸	Dormancy process; phosphorylation; regulation of DNA-templated transcription; regulation of growth; response to antibiotic; single-species biofilm formation

Location P = Chongkrachok Mountain, Prachuap Khiri Khan Province; Location S = Phromawat Temple, Chonburi Province. GO = Gene Ontology. Only major biological processes relevant to antimicrobial resistance are shown to improve table readability. Fold change <1 indicates lower abundance in location P and higher abundance in location S. ABC = ATP-binding cassette.

**Table 3:** Up- and downregulated antimicrobial resistance-associated proteins of Salmonella spp. identified in the fecal microbiomes of long-tailed macaques from location P compared with location S.

**Protein ID**	**Protein name**	**Peptide sequence**	**Fold change**	**p-value**	**Gene ontology (GO) – Biological process**
**Upregulated proteins in location P**					
P37589	Signal transduction protein PmrD (BasR post-transcriptional activator; polymyxin resistance protein PmrD)	KSHYVKK	3.4640	5.23 × 10⁻²⁰	Response to antibiotic
Q9R381	Aminoglycoside N(6')-acetyltransferase type 1 (EC 2.3.1.82) (AAC(6')-Iy)	DIRQMNK	2.9683	1.04 × 10⁻¹⁵	Acetyl-CoA metabolic process; response to antibiotic
**Downregulated proteins in location P**					
P0A255	Aminoglycoside N(3)-acetyltransferase III (EC 2.3.1.81) (AAC(3)-III)	LDDKARR	0.46266	3.90 × 10⁻⁴	Response to antibiotic
Q8XFG0	Multidrug resistance protein MdtM (MDR efflux pump)	FTLFSNNLPK	0.43304	7.45 × 10⁻⁵	Response to antibiotic

Location P = Chongkrachok Mountain, Prachuap Khiri Khan Province; Location S = Phromawat Temple, Chonburi Province. GO = Gene Ontology. Fold change >1 indicates higher abundance in location P, whereas fold change <1 indicates higher abundance in location S. Only biological processes associated with antimicrobial resistance are presented.

Proteins associated with AMR were enriched in the β-lactam resistance pathway. Outer membrane protein TolC from location P was upregulated 2.67-fold and was associated with the resistance-nodulation-division (RND) efflux pump system. KEGG mapping indicated that TolC functions as a MDR-associated protein involved in active antibiotic efflux.

In contrast, proteins enriched in location S included multidrug efflux pump-associated proteins and class D β-lactamases, including AcrA, β-lactamase 1, β-lactamase OXA-7, and β-lactamase OXA-2. These proteins were associated with multidrug efflux mechanisms and β-lactamase-mediated antibiotic degradation.

Notably, habitat-dependent expression of specific resistance-associated proteins was observed. TolC-mediated RND efflux activity predominated in the natural ecotourism habitat (location P), whereas multiple β-lactamases and AcrA-associated efflux mechanisms predominated in the urban-proximate habitat (location S), suggesting distinct AMR strategies across habitats.

## DISCUSSION

### Habitat-associated variation in gut metaproteomic profiles

NHPs are highly relevant animal models for human research. Extensive investigations of their microbial composition have provided critical insights into the factors shaping host–microbiome interactions. Metaproteomics has become a powerful tool for studying the expression and functions of the gut microbiome by analyzing the entire proteome of animal microbial communities. The present study provides novel protein level evidence that habitat-specific anthropogenic interactions shape expressed AMR functions in gut *Escherichia* spp. and *Salmonella* spp., complementing and extending metagenomic findings by identifying mechanisms that are actively translated in macaque populations from Chongkrachok Mountain, Prachuap Khiri Khan Province (location P), and Phromawat Temple, Si Racha District, Chonburi Province (location S). Long-tailed macaques naturally forage on leaves, fruits, and insects; however, individuals at the present sampling sites also relied heavily on anthropogenic food sources, including food waste and food provided by residents and tourists. Location P represents a nature-based ecotourism setting characterized by frequent human–wildlife interactions. In contrast, habitat fragmentation and expanding human activities have pushed macaques closer to urban areas, such as location S, leading to interactions that may have both beneficial and adverse effects on surrounding human communities. In this shared human–macaque landscape, we performed a comprehensive fecal gut microbiome proteomic analysis of wild macaques in Thailand.

The results showed that protein expression patterns of *Escherichia* spp. and *Salmonella* spp. differed markedly between the two locations. A study comparing the microbiomes of wild and captive long-tailed macaques revealed that captivity conditions may alter gut microbiome richness and key metabolic functions [26]. In addition, Muhammad *et al.* [27] found that long-tailed macaque populations receiving anthropogenic food had higher gut microbiota richness and evenness than populations inhabiting different habitats. Further studies have also reported that geographic location and environmental factors are critical determinants of the structure and function of gut microbial communities in animals [28, 29]. Wang *et al.* [29] investigated the role of geographic location and environmental factors in shaping the gut microbiota of Himalayan langurs and Xizang macaques. Differences in the prevalence of *E. coli* and MDR genes were observed among langurs from four locations with different geographic and altitudinal characteristics, suggesting that these animals may serve as reservoirs for antimicrobial-resistant *Escherichia* spp. under unique ecological pressures. Therefore, variation in gut microbial protein expression between macaque populations in the present study was likely driven by differences in dietary patterns, geographic location, and human–macaque interactions. Nevertheless, limited awareness of pathogen transmission among tourists, together with poorly managed human–wildlife interactions, may increase the risk of infectious disease transmission.

### Functional interpretation of DEPs

The present study compared upregulated and downregulated proteins in gut *Escherichia* spp. and *Salmonella* spp. from fecal samples of macaques inhabiting locations P and S. Among the upregulated proteins of *Escherichia* spp., most were involved in translation and DNA-templated transcription. Meanwhile, the most significantly altered biological processes among downregulated proteins in location P were DNA-templated transcription, proteolysis, DNA replication, and response to antibiotics. These functions are closely associated with microbial proliferation. Excessive proliferation of *E. coli* in the intestine may disrupt the gut microbiota and exacerbate inflammation of the large intestine [30].

Among *Salmonella* spp., most of the upregulated proteins expressed at location P were involved in DNA repair, the SOS response, and enterobacterial common antigen biosynthesis. Proteins encoded by SOS-associated genes are involved in DNA repair and maintenance of crucial cell division proteins [31]. These shifts in proliferation- and repair-related proteins provide functional evidence of microbial adaptation to anthropogenic pressures and extend beyond taxonomic shifts reported in previous Thai macaque *16S rRNA* studies. Meanwhile, downregulated DEPs were involved in cobalamin biosynthesis, methylation, and glutamine metabolism. A previous study using a metaproteomic approach to examine microbial protein expression during a controlled dietary intervention revealed significant metabolic alterations in microbial responses to different dietary patterns [32]. Thus, metaproteomics is a valuable approach for investigating microbial metabolism associated with diet and its potential influence on host health.

### Antibiotic resistance-associated proteins and anthropogenic influence

Interestingly, the present study revealed that proteins associated with AMR responses in *Escherichia* spp. were more abundant at location S than at location P. The macaques in location S inhabited an urban-proximate temple environment with frequent human contact. Previous studies in India indicated that environmental AMR pollution resulting from human activity is a long-standing concern. Evidence from medicinal plants, hospital effluents, and untreated wastewater showed that hospital effluents contained *E. coli* resistant to extended-spectrum cephalosporins and fluoroquinolone antibiotics [33, 34]. In addition, a study on AMR and the epidemiology of *E. coli* isolated from town hospitals in Shandong Province, China, reported that colonization with extended-spectrum β-lactamase-producing *E. coli* increased by 62.8% among outpatients across three regions of Shandong Province. These rates were considered to reflect contact with food-producing animals in rural areas [35].

The present study examined molecular mechanisms of resistance to β-lactam antibiotics using a metaproteomic approach and demonstrated that the fecal microbiome may respond differently according to habitat-associated dietary and environmental exposures. β-lactam antibiotics, including penicillins, cephalon-sporins, carbapenems, monobactams, and penems, are among the most widely used drugs for treating bacterial infections [36]. These antibiotics primarily inhibit bacterial growth by disrupting bacterial cell wall synthesis, reducing selective permeability, causing cell death, and inactivating penicillin-binding proteins associated with cell wall synthesis [37, 38]. According to KEGG pathway analysis of upregulated antibiotic resistance-associated proteins of *Escherichia* spp. from long-tailed macaque fecal microbiomes, TolC from location P was upregulated in the β-lactam resistance pathway. This protein was associated with MDR through the RND efflux pump mechanism. Previous research reported that RND family transporters are widespread, particularly among Gram-negative bacteria, and actively efflux many antibiotics and chemotherapeutic agents [39]. The RND family includes several members relevant to antibiotic resistance in *Escherichia* spp. and typically consists of homomeric assemblies comprising an inner membrane pump, a membrane fusion protein, and an outer membrane porin [40]. A distinctive characteristic of RND efflux pump systems is that they span both the inner and outer bacterial membranes. Their function is to transport antibiotics out of the bacterial cell, preventing the drugs from reaching their intended targets. These systems also play important roles in bacterial physiology, pathogenicity, and metabolism.

Regulation of the RND efflux pump remains incompletely understood. Previous literature indicates that the RND efflux system in enteric bacteria is controlled by natural inducers encountered during infection, such as bile salts and fatty acids [41]. Previous studies have also reported that activation may occur through the inactivation of local repressors that block the expression of structural pump genes, such as AcrR, or through the activation of global transcriptional regulators, such as SoxS, RobA, or RamA [42, 43]. In addition, metal cations, which act as cofactors in several bacterial processes, may induce the expression of RND efflux pumps. In particular, studies on the metal-induced regulation of CusCBA proteins, members of the RND protein superfamily of proton-driven cation symporters and antiporters in *E. coli*, have shown that copper and silver ions may serve as natural inducers of CusCBA expression [44, 45].

In location S, AcrA and class D β-lactamases, including β-lactamase 1, β-lactamase OXA-7, and β-lactamase OXA-2, were upregulated and involved in multidrug efflux and β-lactamase enzyme activity. When exposed to antibiotics, bacteria often overexpress efflux pumps or accumulate mutations, especially in regulatory genes, allowing them to expel antibiotics more efficiently. Previous studies have shown that constitutively expressed efflux pumps often act in concert with other resistance mechanisms, such as β-lactamases, to confer antibiotic resistance [46–48]. Moreover, a review reported that multidrug efflux pumps are involved in several cellular processes, including biofilm development, and play a pivotal role in regulating specific genes and adaptation to environmental conditions [49]. The production of β-lactamases is the most common mechanism of resistance to β-lactam antibiotics in Gram-negative bacteria [50]. Evidence of extended-spectrum β-lactamase-producing *E. coli* in the gut microbiomes of wild animals indicates that dissemination in wildlife is increasing worldwide [51–53]. Therefore, β-lactam resistance in Gram-negative bacteria requires careful monitoring to support the development of effective control and mitigation strategies.

### Wildlife as sentinels of environmental AMR

Previous research suggests that wild NHPs are generally not directly exposed to antibiotics but may acquire antibiotic-resistant, β-lactamase-producing *E. coli* through foraging and drinking from natural environments contaminated by anthropogenic sources [54]. Moreover, agriculture and livestock activities, together with lifestyle changes in free-living NHPs transitioning to captive or semi-captive conditions, may increase human–NHP interactions and enhance the spread of antibiotic-resistant bacteria [55]. As shown in the present study, expression of antibiotic resistance-associated proteins and β-lactam resistance mechanisms among *Escherichia* spp. from location S appeared more complex than those observed in location P. Location P, Chongkrachok Mountain, represents a nature-based ecotourism site with increasing interactions between humans and wild macaques, suggesting that tourism may intensify rather than reduce human–macaque conflict. Such interactions may reduce natural foraging success, increase reliance on anthropogenic foods, and contribute to the emergence of antibiotic-resistant *E. coli* strains. In contrast, location S, Phromawat Temple, represents an urban-proximate habitat where macaque populations interact frequently with human communities. Habitats near human activity have been shown to harbor greater diversity of antimicrobial-resistant genes in NHP *E. coli* isolates, indicating that macaques may have substantial potential to spread MDR [56]. By demonstrating elevated expression of β-lactam resistance proteins in urban-proximate macaques, the present metaproteomic data provide functional support for wildlife as sentinels of environmental AMR pollution, offering insights that gene-centric studies alone cannot provide and informing tourism management and surveillance strategies in Thailand.

### One Health implications and future perspectives

Compared with other microbiome approaches, metaproteomic data on wildlife resistomes remain limited, particularly for Southeast Asian NHPs. Beyond microbiome monitoring, metaproteomics can estimate microbial abundance and molecular processes relevant to the One Health framework, supporting the development of effective gut microbiome control algorithms [57, 58]. The present findings suggest that *M. fascicularis* populations are influenced by anthropogenic activities that alter their foraging strategies and diets, particularly their access to human-derived foods. Human–macaque interactions were more common in urban habitats. Nevertheless, inducers of antibiotic resistance are complex and likely reflect environmental cues specific to each ecosystem. Environmental pollution associated with human activities and the use of antimicrobial compounds may significantly shape gut microbiota expression and negatively affect wildlife conservation and natural ecosystems. Therefore, the present findings provide important insights into how habitat, diet, and geographic variation affect AMR expression and resistance mechanisms, supporting the adoption of the One Health approach in AMR surveillance. Overall, this study improves understanding of the role of wild animals in AMR dissemination, supports the development of effective control measures to minimize transmission risks from antibiotic-resistant gut microbiota, and contributes to estimating the emergence and spread of zoonotic and anthroponotic diseases at the human–wildlife interface.

## CONCLUSION

This study provides the first shotgun metaproteomic characterization of habitat-associated gut resistomes in free-living *M. fascicularis* from Thailand. Distinct protein expression profiles were observed between macaque populations inhabiting natural ecotourism and urban-proximate environments, demonstrating that habitat characteristics and anthropogenic influences can shape the functional expression of AMR-associated proteins within the gut microbiome. A total of 1,299 DEPs were identified in *Escherichia* spp. and 231 DEPs in *Salmonella* spp., highlighting substantial habitat-related differences in microbial activity. Functional analyses revealed that proteins associated with microbial proliferation predominated in *Escherichia* spp., whereas DNA repair and stress-response functions were more prominent in *Salmonella* spp. from location P. Notably, *Escherichia* spp. from location S exhibited a greater abundance and diversity of antibiotic resistance-associated proteins, including multidrug efflux components and β-lactamase-related mechanisms.

From a practical perspective, these findings suggest that free-living macaques inhabiting environments with greater human influence may serve as important reservoirs and sentinels for the dissemination of environmental AMR. The identification of habitat-specific resistance mechanisms provides valuable information for wildlife health monitoring, environmental surveillance programs, and One Health-based AMR management strategies. Furthermore, the results emphasize the importance of managing human–wildlife interactions, anthropogenic food provisioning, and environmental contamination to reduce the potential spread of antimicrobial-resistant microorganisms at the human–animal–environment interface.

A major strength of this study is the application of shotgun metaproteomics, which enabled direct assessment of actively expressed resistance-associated proteins rather than merely detecting the presence of resistance genes. This approach provides a more biologically relevant understanding of microbial functional responses to environmental pressures. However, the study was limited by its relatively small sample size, restricted geographic coverage, pooled-sample design, and the lack of complementary metagenomic, metabo-lomic, and environmental exposure data. Consequently, causal relationships between habitat characteristics, dietary factors, and AMR expression could not be fully established.

Future studies should incorporate larger multicenter sampling schemes, longitudinal monitoring, dietary profiling, environmental contaminant assessments, and integrated multi-omics approaches to elucidate the ecological drivers of AMR expression in wildlife populations. Comparative investigations involving additional primate species and habitats would further enhance understanding of wildlife-associated resistome dynamics.

Overall, the present findings demonstrate that habitat-associated anthropogenic pressures influence the functional expression of AMR-associated proteins in the gut microbiota of free-living long-tailed macaques. These results advance current knowledge of wildlife resistome ecology and reinforce the importance of incorporating wildlife populations into One Health surveillance frameworks aimed at mitigating the emergence and spread of AMR.

## DATA AVAILABILITY

The raw MS/MS spectral data are available on the ProteomeXchange repository under registration numbers JPST004417 (https://repository.jpostdb.org/preview/10564982166993c16aa833d; Access key:8452) and PXD074529.

## GENERATIVE AI DECLARATION

The authors declare that generative artificial intelligence (AI) tools were used solely to improve language, grammar, and readability during manuscript preparation. All scientific content, data analysis, interpretation of results, and conclusions were developed and verified by the authors. The authors take full responsibility for the accuracy, integrity, and originality of the work presented, and no AI tool was listed as an author.

## AUTHORS’ CONTRIBUTIONS

WF: Conceptualization, investigation, methodology, supervision, and writing—review and editing. DT: Investigation, supervision, and validation. SC and NP: Methodology. SR: Data curation, investigation, methodology, and writing—review and editing. KP: Data curation, formal analysis, methodology, validation, and writing—original draft. All authors have read and approved the final manuscript.
